# Consideration of a metabolic disorder in the differential of mild developmental delay: A case of nonketotic hyperglycinemia revisited 36 years later

**DOI:** 10.1002/jmd2.12208

**Published:** 2021-03-31

**Authors:** Timothy F. Tramontana, Theodore E. Wilson, Bryan E. Hainline

**Affiliations:** ^1^ Department of Medical and Molecular Genetics Indiana University School of Medicine Indianapolis Indiana USA

**Keywords:** *AMT*, *GLDC*, glycine cleavage enzyme system (GCS), nonketotic hyperglycinemia (NKH)

## Abstract

We present a 53‐year‐old male with nonketotic hyperglycinemia (NKH) who presented in decompensated state to our university hospital several months prior to a primary diagnosis of multifocal pneumonia accompanied by reports of seizure‐like activity, altered mental status, tremors, and fever. He was initially diagnosed with NKH in his preschool years, over 40 years previously, along with his younger sister. At that time, he had developmental and physical delays (which his sister also experienced). His health course has been relatively uneventful otherwise, as regards decompensation of his disease, and he has not been on the standard regimens of reduced dietary glycine intake along with dextromethorphan and sodium benzoate. Recent molecular confirmation of NKH was completed and both he and his sibling likely have an attenuated form of NKH mediated by the combined effects of their variants. This paper presents what we believe to be report of the oldest surviving individuals with attenuated NKH.

## 1 INTRODUCTION

Nonketotic hyperglycinemia (NKH) is a disorder of deficiency of the glycine cleavage enzyme system (GCS). It is an autosomal recessive inborn error of metabolism which often results in early death. The disease is characterized by genotypic and phenotypic heterogeneity. Two genes associated with the classic form of NKH include *GLDC* and *AMT*. Patients with absence of the GCS typically present during the neonatal period with symptoms including microcephaly, poor muscle tone, seizures, lethargy, and apnea necessitating transient ventilatory support. Many patients succumb to the disease during this time period. Those who survive the initial period are left severely developmentally and intellectually delayed. Seizures typically worsen and as a result of poor airway maintenance (secondary to hypotonia) many succumb to respiratory issues in early childhood.

The attenuated form of NKH has a more mild and heterogeneous course. It is typically characterized by a combination of variable developmental progress +/− epilepsy. Intermittent manifestations of attenuated NKH underscore the importance of thorough evaluation of patients with transient neurologic dysfunction.^1^ Patients with intermittent neurologic dysfunction associated with fever and characterized by movement disorder, especially when accompanied by gaze palsy, should have testing for NKH.^1^ Attenuated NKH can be subdivided into attenuated poor, intermediate, and good categories based on the degree of developmental delay and presence or absence of epilepsy.^2^ Variable developmental outcomes are thus the norm for this form of NKH. Individuals may need to attend special education classes and some may attend mainstream classes. Choreoathetoid movements may be present at baseline, but in some cases may occur as a result of a febrile illness. In addition, febrile illness can result in lethargy, altered mental status, agitation, chorea, seizures, and intellectual disability.

### 1.1 Case

The patient is a 53‐year‐old male with a history of NKH. His biochemical/clinical diagnosis was initially made as a toddler.^3^ This evaluation was initially prompted by a history of physical and developmental delays as well as occasional choreoathetosis. It should be noted that his sister (26 months his junior) also had similar delays prompting further study. Urine chromatography completed on his sister at 35 months of age showed an abnormally high glycine level. In addition to routine urine and hematologic studies (which were normal), a glycine loading study was completed on these patients. Pretest and posttest glycine levels were found to be elevated in CSF, urine, and serum of both patients. Glycine/serine interconversion was also impaired as well as an abnormal electroencephalographic pattern following glycine administration.^3^ Based on both biochemical and clinical phenotype at the time, the diagnosis of NKH was made.^4^ These patients fell into the attenuated classification of NKH with developmental and physical outcomes much less severe than in the classic type. Follow‐up evaluations over the next 6 years showed continuation of developmental delays with a Wechsler Intelligence Scale Revised (WISC‐R) of 76 at 9 years 3 months.^3^ At the time of diagnosis, the patient was given no form of treatment other than a low‐protein diet. Additionally, he was not assigned regular follow‐up as this condition at the time was not considered treatable, which was a recommendation carried forward for 36 years. Since diagnosis, he has had no history of seizures, but has had persistent cognitive delay. The patient has lived independently for the majority of his life and has not followed a protein restricted diet nor been on pharmacologic therapy.

There have been two reported episodes of metabolic decompensation over the last 2 years. Both were similar in nature with episodes of shaking, worsening ataxia, and fever. At that time, CSF studies showed elevated glycine levels. Gradual recovery occurred without pharmacologic or dietary intervention, although he reports increased tremors and decreased mobility since that time. His most recent admission was in July 2019. He was reported to have started with tremor and dystonia along with confusion and fever. He was presented with tremulousness that was thought to be seizures and required four point restraints to prevent him from falling off the bed. This progressed to needing intubation. Multifocal pneumonia was identified on chest x‐ray and antibiotic treatment started. Plasma amino acids obtained during admission and prior to treatment revealed a glycine level of 482 mcmol/l (range: 129‐479). Repeat plasma amino acids on treatment and nutritional repletion showed a glycine level of 444 mcmol/L. MRI and CT imaging were unremarkable. He was started on dextromethorphan at 150 mg twice daily and sodium benzoate at 5.5 g 4 times daily (which was eventually stopped in October 2019 due to concerns over worsening tremor). Extubation occurred approximately a week after admission, but worsening of cardiorespiratory status prompted reintubation and transfer to our university hospital for subspecialty care. During this hospital admission, the patient continued to have diffuse myoclonic jerks necessitating neurology consult for further treatment strategies. On EEG these were not epileptic. The patient was eventually extubated and transferred to a rehab facility a month after initial admission. Recommendations going forward were for treatment with dextromethorphan/sodium benzoate and a 40 to 50 g per day protein restriction.

At his 6 month follow‐up in January 2020, the patient continued to have issues with cognition and recall. He also has issues with tremor, myoclonic jerks, and ataxia since discharge. He is currently on pharmacologic treatment (dextromethorphan) and trying to restrict his protein intake. His myoclonic jerks and tremors seem to be fairly well controlled on clonazepam. His plasma amino acids in January 2020 when off sodium benzoate for approximately several months was 563 mcmol/L.

### 1.2 Genetic findings

Given the previous diagnosis of NKH without genetic confirmation, a testing strategy was chosen to investigate genes associated with NKH. Investigation included those associated with both classic and attenuated forms of NKH. The gene panel included *ALDH7A1*, *AMT*, *BOLA3*, *GCSH*, *GLDC*, *GLRX5*, *HCFC1*, *IBA57*, *ISCA2*, *LIAS*, *LIPT1*, *LIPT2*, *NFU1*, *PLPBP*, *PNPO*, and *SLC6A9*. The results of XomeDx Slice (GeneDx, Gaithersburg, Maryland) included two likely pathogenic variants in the *GLDC* gene. The variants included: p.Arg739His (CGC>CAC):c.2216G>A in exon 19 (NM_000170.2) and c.2919+5G>T:IVS24+5G>T in intron 24 (NM_000170.2). The p.Arg739His variant has been identified in patients with NKH in published literature.^5^ Functional studies for this variant showed decreased enzyme activity at 6% to 8% of normal *GLDC* activities.^6^ In addition, it has not been observed at significant frequency in large population cohorts.^7^ The intronic variant has been observed in homozygous state before in a patient with classic NKH.^8^ This splice site variant destroys the canonical splice donor site in intron 24. It is predicted to cause abnormal gene splicing resulting in an in‐frame protein product with an abnormal message.^9^ In addition, it has not been observed at significant frequency in large population cohorts.^7^

*GLDC* is a gene located on chromosome 9 that codes for a protein called glycine decarboxylase. It forms a critical component of the GCS necessary for the conversion of glycine to serine. The GCS is confined to the mitochondria and is composed of four protein subunits including a P, H, T, and L proteins. The P protein is the pyridoxal phosphate dependent glycine decarboxylase. Its function is to cleave glycine, which in addition to its role as an amino acid, plays an important role as a neurotransmitter critical to the proper functioning and development of the central nervous system. Significant loss of enzyme activity is associated with ubiquitous elevations of glycine (most importantly in the brain) which is neurotoxic and leads in its most severe form to symptoms including breathing difficulties, intellectual disability, developmental delays, and intractable seizures.

Biallelic pathogenic variants in *GLDC* are typically associated with classic NKH which is at the severe end of the NKH spectrum. The array of genes in the custom exome slice included those that were associated with milder forms of NKH as our patient (and his affected sibling) clearly followed a more benign course than is typically associated with variants in the classic forms including both the *GLDC* and *AMT* genes. The presence of one variant with residual activity is necessary, but not sufficient for attenuated outcome.^10^ Enzyme activity levels as low as 1% is sufficient enough to result in an attenuated presentation. The intronic variant predicted to destroy the natural donor splice site and observed in homozygous state in a patient with classic NKH likely results in complete or near‐complete loss of function (ie, <1% residual function). The missense variant likely resulted in residual enzyme activity at a level sufficient to result in an attenuated phenotype seen in both this patient and his sister.

## 2 CONCLUSION

NKH is an autosomal recessive disease which exists in both the classic (severe) and attenuated forms. It can result from mutations in one of three different genes, although a small percentage of cases do not have an identifiable pathogenic variant in one of those genes. The attenuated form increases in prevalence with later the onset of symptoms. Attenuated NKH can be broken down into three categories based on developmental progress and the presence or absence of epilepsy.^1^ Individuals in the attenuated poor category have developmental quotients (DQ) <20 and all have epilepsy.^1^ The attenuated intermediate category results in a DQ of 20 to 50 with easily treatable or no epilepsy.^1^ Finally, those in the attenuated good category have a DQ > 50 and do not have epilepsy.^1^ Our patient falls into this third category. This case illustrates a few important points about this disorder. First, it is a disease process in which treatment is both dietary and pharmacologic. Early treatment with dextromethorphan and sodium benzoate has been found sufficient to normalize plasma glycine levels and is effective at improving outcome if used in children with attenuated disease with mutations providing residual activity and when started from the neonatal period.^11^ It is important to note though that patients with attenuated NKH and residual enzyme activity in whom protein restriction and pharmacologic treatment (with sodium benzoate) are used should be monitored closely as suboptimal glycine levels can be associated with sodium benzoate toxicity. Second, pathogenic variants typically associated with a severe form can result in attenuated phenotype as in our patients. Two likely pathogenic variants in the *GLDC* gene, which are typically associated with the classic NKH presentation of early and severe onset, resulted in an attenuated phenotype in our patient and his sister. Third and most important is the subtle developmental issues patients may have with the attenuated phenotype that may not raise suspicion for this disease and result in either delayed or insufficient testing. Our patient and his sibling illustrate issues involved with all three of these concerns. They had an attenuated phenotype at the time of diagnosis for which our patient has not been treated with consistent therapy and as such has developed a less than optimal disease phenotype. Depending on the patient's genotype and the degree of residual enzyme activity early and aggressive treatment can result in positive outcomes. At the present time, as far as the authors are aware, the patient's sister (who is not established in our clinic) is living out of state and has neurocognitive issues like her brother absent the spasticity and tremulousness that has affected him.

Individual homozygous for p.Ala802Val (which is associated with substantial residual GCS activity) who received early and aggressive treatment in the first 2 years of life had normal intelligence.^12^ In addition, both he and his sister have a combination of variants typically associated with the severe form, but ultimately resulting in an attenuated phenotype. They also had a subtle developmental phenotype which certainly could have resulted in them not being diagnosed with this disease as a result of provider's inexperience with such patients. This case emphasizes the need for longitudinal follow up of cases identified even 36 years ago.

## CONFLICT OF INTEREST

Timothy Tramontana, Theodore E Wilson, and Bryan Hainline declares they have no conflict of interest.

## PATIENT CONSENT

Informed consent was obtained from all patients for being included in the study.
